# Ηand dexterities assessment in stroke patients based on augmented reality and machine learning through a box and block test

**DOI:** 10.1038/s41598-024-61070-x

**Published:** 2024-05-08

**Authors:** Georgios Papagiannis, Αthanasios Triantafyllou, Konstantina G. Yiannopoulou, George Georgoudis, Maria Kyriakidou, Panagiotis Gkrilias, Apostolos Z. Skouras, Xhoi Bega, Dimitrios Stasinopoulos, George Matsopoulos, Pantelis Syringas, Nikolaos Tselikas, Orestis Zestas, Vassiliki Potsika, Athanasios Pardalis, Christoforos Papaioannou, Vasilios Protopappas, Nikolas Malizos, Nikolaos Tachos, Dimitrios I. Fotiadis

**Affiliations:** 1 Biomechanics Laboratory, Physiotherapy Department, University of the Peloponnese, 23100 Sparta, Greece; 2 Physioloft, Physiotherapy Center, 14562 Kifisia, Greece; 3 Department of Physiotherapy, University of West Attica, 12243 Athens, Greece; 4 Sports Excellence, 1St Department of Orthopaedic Surgery, National and Kapodistrian University of Athens, 12462 Athens, Greece; 5 Biomedical Engineering Laboratory, National Technical University of Athens, 9, Herοon Polytechniou Str., Zografou, 15773 Athens, Greece; 6 CNA Lab, Department of Informatics, Telecommunications University of Peloponnese, 22100 Tripoli, Greece; 7 Unit of Medical Technology and Intelligent Information Systems, University of Ioannina, 45110 Ioannina, Greece; 8 Ostacon Ltd, 167 77 Elliniko, Greece; 9 Biomedical Research Institute, Foundation for Research and Technology-Hellas (FORTH), 70013 Heraklion, Greece

**Keywords:** Neurological disorders, Rehabilitation, Software

## Abstract

A popular and widely suggested measure for assessing unilateral hand motor skills in stroke patients is the box and block test (BBT). Our study aimed to create an augmented reality enhanced version of the BBT (AR-BBT) and evaluate its correlation to the original BBT for stroke patients. Following G-power analysis, clinical examination, and inclusion–exclusion criteria, 31 stroke patients were included in this study. AR-BBT was developed using the Open Source Computer Vision Library (OpenCV). The MediaPipe's hand tracking library uses a palm and a hand landmark machine learning model to detect and track hands. A computer and a depth camera were employed in the clinical evaluation of AR-BBT following the principles of traditional BBT. A strong correlation was achieved between the number of blocks moved in the BBT and the AR-BBT on the hemiplegic side (Pearson correlation = 0.918) and a positive statistically significant correlation (*p* = 0.000008). The conventional BBT is currently the preferred assessment method. However, our approach offers an advantage, as it suggests that an AR-BBT solution could remotely monitor the assessment of a home-based rehabilitation program and provide additional hand kinematic information for hand dexterities in AR environment conditions. Furthermore, it employs minimal hardware equipment.

## Introduction

Nearly 80% of stroke patients suffer from upper extremity disabilities, such as unilateral paresis and sensory abnormalities^1^. Such disorders might cause reduced hand function, leading to exercise restriction and engagement behavioral inhibition^2^. Most of the patients recover gradually from such deficiencies, frequently attaining maximal recovery after 6–12 months of treatment^3^. It is recommended that patients are frequently evaluated at multiple time periods to review prognoses and design treatment strategies. Therefore, experts advocate regular monitoring and evaluation using validated and objective markers^4^.

**Figure 1:**
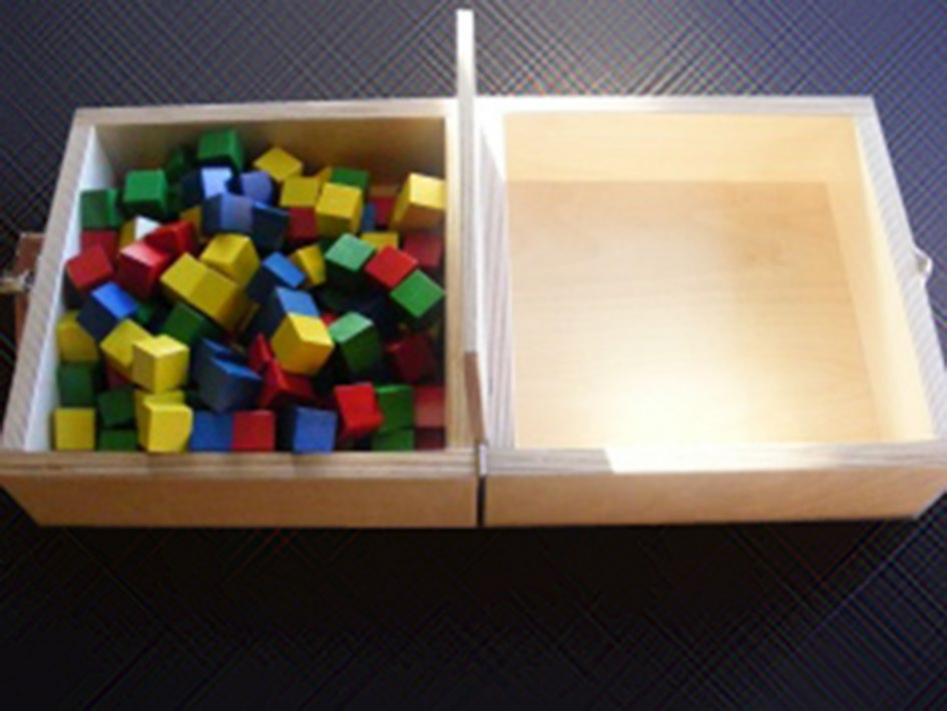
The original box and block test.

A popular and widely accepted measure for assessing unilateral hand motor skills is the box and block test (BBT)^5,6^. Its original form consists of a box with two compartments separated in the middle by a barrier and 150 cubes of approximately 1 in^3^ each. The test uses the same hand to score the highest possible number of cubes from the compartment of the examined hemiparetic side to the opposite (horizontal adduction movement of the examined arm (Fig. 1). The number of accurately relocated blocks within 60 s determines the test's score. The BBT has demonstrated excellent inter- and intra-examiner reliability and represents a valid procedure for patients suffering from neurological disorders^6–11^.

As the stroke rate rises, neurorehabilitation and developing innovative, efficient treatment approaches utilizing virtual reality (VR) and augmented reality (AR) solutions are increasingly important. AR can facilitate the delivery of goal-oriented activities, sensorimotor and performance feedback, and enhanced intervention results^12–15^. Several studies have revealed that AR/VR might increase patient compliance by promoting their motivation^16,17^. Including entertaining parts in the treatment, such as those provided by serious games, can be valuable in this direction^18^. Moreover, when equipped with an autonomous headgear, VR allows patients to execute their treatment at home. Most AR/VR systems can monitor hand motions using infrared cameras or inertial measurement unit-integrated controllers. The use of fully immersive VR technology to perform functional evaluation and analyze upper extremity kinematics in post-stroke rehabilitation^2^ has not been investigated yet.

There is high scientific reasoning behind the development of AR tests. Using the automatic computing of quantitative measures, it may be possible for healthcare and rehabilitation professionals to measure the patient's performance objectively, thus reducing the inter-rater variability encountered in the conventional assessment. It may also be a component of a complete home-based virtual evaluation process that patients might utilize to extract objective information on their improvement, providing clinicians with valuable input to adapt treatment regimens^4^. In response to these demands and the rising popularity of telehealth, the development of such VR/AR systems has the potential to serve as an objective clinical assessment tool.

The exploration of virtual BBT started less than a decade ago when Chih-Pin Hsiao et al.^19^ developed a digital BBT using a depth-sensing camera, an existing wooden box and blocks, and a host computer. They aimed to detect and record hand kinematics data to provide clinicians with additional applications of VR-BBT to the test's score.

Two years later, Cho et al.^20^ employed non-immersive virtual reality equipment and a depth-sensing sensor to design a VR-BBT to evaluate hand and finger dexterity and validate its efficiency in patients suffering from stroke. Comparing traditional and virtual BBTs demonstrates the viability of converting traditional and unsupervised assessments into a virtual setting. Despite the significant correlations between conventional and VR-BBT in the relative score comparison of affected and non-affected sides, VR-BBT scores were lower than BBT scores. Cho et al. noted that the absence of an actual object is an essential factor that impacts the usability and convenience of virtual reality interactions due to the lack of physical feedback, resulting in different data compared to the real world. Nevertheless, the strong connection between the two assessments is considered significant for their clinical use and subsequent research. However, this approach requires expensive equipment that is not portable and does not accurately visualize the patient's hand,

Ona et al. examined the computerization of the BBT scoring and the automatic administration of the test in 2017^21^ by combining the traditional BBT instrument with the information obtained from a KinectR depth sensor to manage the counting of blocks, as well as acquiring supplementary measurements regarding the subject's movements. They utilized a user interface to allow the clinician to perform the test locally or remotely, retain the collected data, gain access to an up-to-date database structure, and create a report of prior assessments. Their results demonstrated a discrepancy in the number of blocks moved in the real and virtual settings; however, the correlation between the results obtained with the two systems was statistically significant. Additionally, the virtual test's test–retest examination presented a strong and statistically significant correlation. In 2020 Ona et al. proposed a comparable immersive VR implementation on BBT, utilizing controllers, such as the leap motion controller^22^, to evaluate the hand dexterity of Parkinson's disease patients, demonstrating excellent reliability of the measurements. The same year, Rodríguez et al.^23^ assessed hand dexterity in spinal cord injury (SCI) patients and showed a Leap Motion Controller-based virtual version of the BBT. A significant correlation was also found between the actual and virtual versions of the test.

In 2022, using an Oculus Quest controller6, Everard et al. created an immersive virtual version of the BBT (VR-BBT) and assessed its validity among post-stroke and healthy individuals. They discovered significant correlations between the BBT and BBT-VR scores, excellent test–retest reliability, and nearly excellent usability. However, they did observe a statistically significant decline in the VR-BBT score with respect to the BBT.

Augmented reality systems can continually record a wide range of valuable data, such as interactive joint ranges of motion and test scores. In AR, the perceived reality is merged with computer-generated material varying from textual representations to animated digital objects. This provides the opportunity to establish quantitative and objective metrics that may be utilized independently and without the presence of a physician. Several tests have been developed to evaluate the upper limb capabilities of stroke survivors, including motor function^24^ and fine motor skills in a virtual environment^22,25^. In addition to a personal computer, the aforementioned solutions typically require typically costly devices.

Our study aimed to create an augmented reality-enhanced version of the BBT (AR-BBT) using only a laptop and assess its correlation with the original BBT for stroke patients. The research hypothesis states that stroke patients' AR-BBT and BBT scores will exhibit a positive correlation. Thus, our research team developed an AR-BBT system and compared its efficiency with the conventional test. This evaluation was conducted in a clinical study component aimed at creating a comprehensive platform to enhance hand rehabilitation in individuals suffering from a neurological disease.

## Methods

### Development of the AR-BBT

The system's front end comprises a web application based on Microsoft's Active Server Pages Network Enabled Technologies (ASP.NET) and Blazor frameworks, with a Structured Query Language (SQL)-compatible database at its back end. The web application supports basic user and patient management and displays, manipulates, and manages the results received from the system's BBT scores and exercise standalone applications.

#### Augmented reality approach

Our augmented reality box and block test (AR-BBT) improves upon the original BBT by creating an entirely virtual procedure that does not require specialized computer peripherals or high computing power. It only needs a personal computer and a camera, making it portable and light. The system utilizes cutting-edge computer vision and image recognition algorithms, which is an effective standalone application^25,26^. Placing the camera approximately 50 cm away from the hand and providing adequate lighting, the system can accurately monitor the motion of the patient's hand and finger joints.

#### Utilized software modules

The development of the AR-BBT involved utilizing two essential technologies, namely the Open Source Computer Vision Library (OpenCV)^27^ and the ML Hands solution provided by MediaPipe^28^. These technologies played distinct roles in creating an interactive and efficient user experience. OpenCV, a versatile computer vision library, served as the backbone for AR-BBT. It diligently processed each captured frame in nearly real-time, with processing times averaging from 30 to 40 ms per frame. This efficient processing allowed the application to generate virtual elements seamlessly, contributing to the overall smooth and responsive user interface. On the other hand, MediaPipe Hands, an advanced machine learning solution, offered a robust model and pipeline architecture specifically designed for accurate and rapid hand estimation. By leveraging machine learning, MediaPipe Hands facilitated the quick and precise tracking of hands and individual digits within the frames. Both technologies employed were meticulously chosen to guarantee efficient processing times, precise outcomes, and seamless integration. Furthermore, both libraries are designed to function across multiple operating systems, thereby inherently embedding cross-platform compatibility within the AR-BBT system without requiring additional implementation practices. This technological approach was crucial in ensuring the accuracy and reliability of hand movement recognition in the AR-BBT.

**Figure 2:**
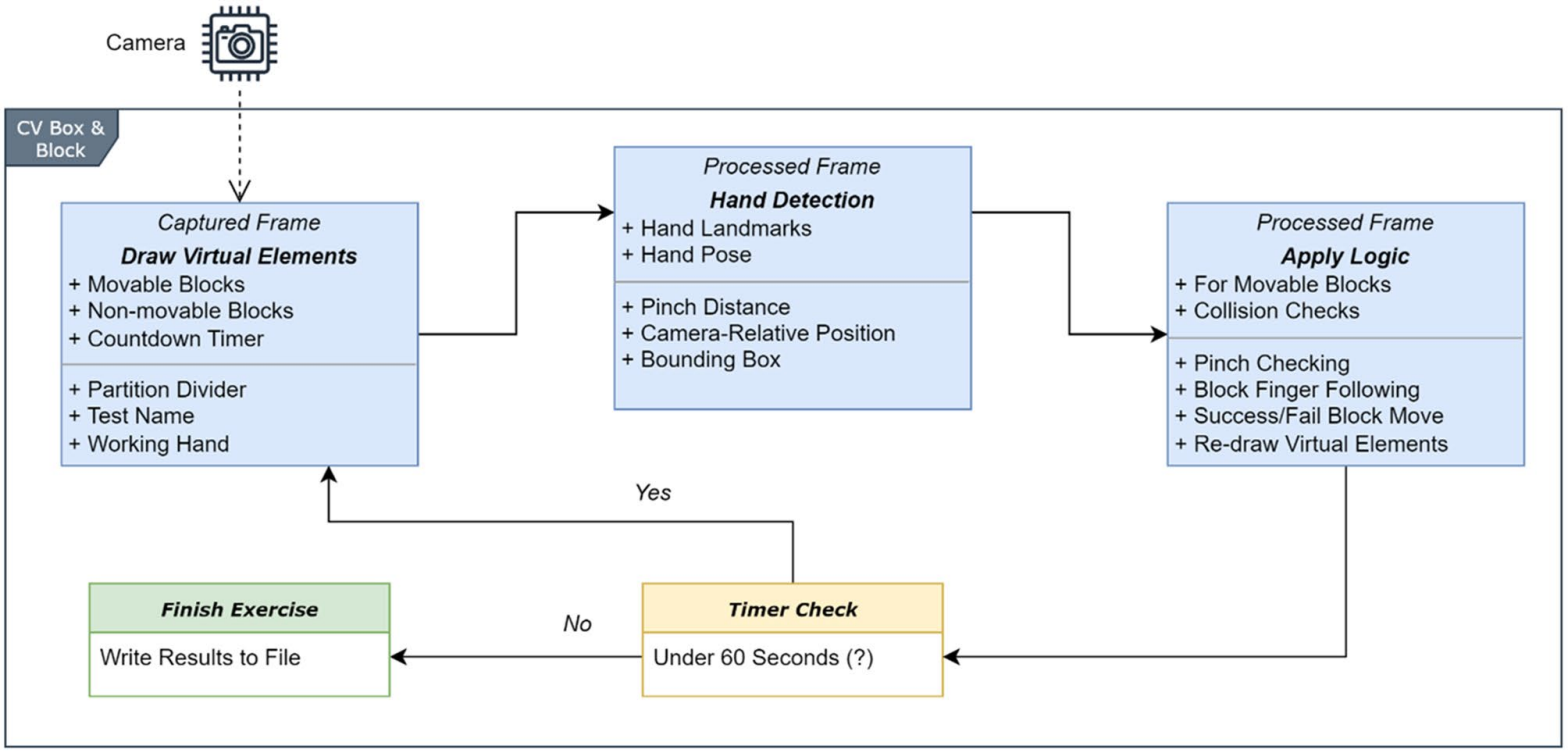
High-level proposed AR-BBT architecture.

The developmental journey of the AR-BBT unfolded in three distinct phases, each contributing to the application's functionality and effectiveness. The first phase involved establishing a robust computer vision environment, creating a solid foundation for subsequent stages. The second phase revolves around the intricate process of detecting and tracking hands and fingers within the captured frames. This issue was critical as it formed the core interactive element of the application. Finally, the third phase encompassed the implementation of operation logic to synchronize the application's response with each frame, ensuring a seamless and engaging user experience. Visualizing the high-level approach of AR-BBT is made possible through Fig. 2, which encapsulates the various elements and interactions that collectively bring the application to life. This diagram serves as an illustrative guide, showcasing how the integration of OpenCV and MediaPipe Hands culminates in creating an innovative and interactive augmented reality box and block test (AR-BBT) application.

#### Modeling the virtual environment

**Figure 3:**
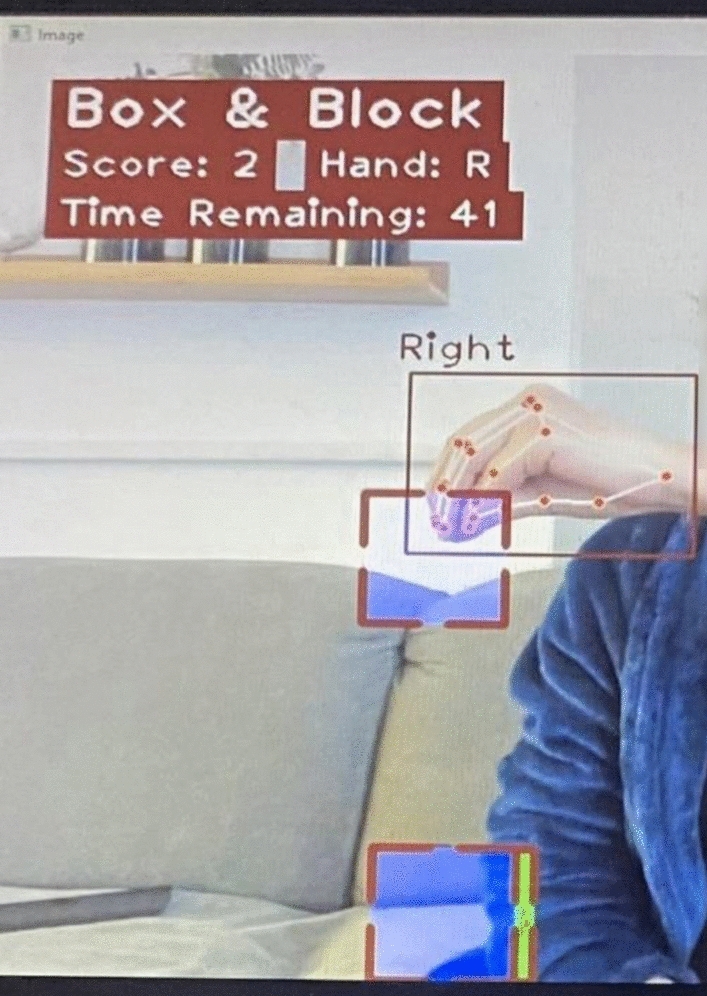
AR-BBT used by a patient suffering from stroke.

To configure the augmented reality environment for the AR-BBT, a single RGB camera captures repetitive frames that are passed into the processing algorithm. A 60 frames per second (fps) camera with a 720p resolution (standard high-definition display resolution of 1280 × 720 pixels) is used for testing, and OpenCV is used to flip the image horizontally, followed by a check for vertical-axis symmetry using horizontal shift and absolute difference computation^27^. The display depicts essential elements such as the session's name, clock, score counter, and divider. To prevent screen clutter, each of the 150 blocks is generated individually, and a successful move occurs when a cube is transferred from one compartment to the other without contacting the partition separator. Each cube's color can be red, green, blue, or yellow, and a transparency filter is applied so that the background remains visible and the patient's view of their movements unobstructed. Successfully transferred blocks persist between frames, encouraging positive reinforcement (Fig. 3).

#### Machine learning hand detection

**Figure 4:**
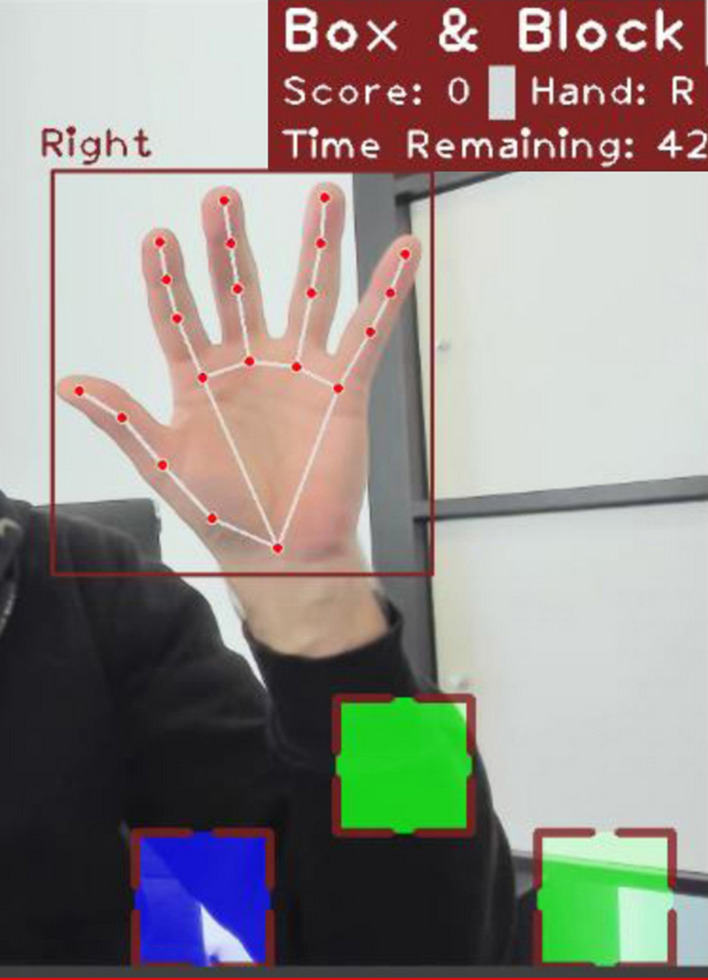
MediaPipe's hand tracking library—hand landmark mode and basic components information at the upper left of the test image.

The MediaPipe's hand tracking^28^ library uses two machine learning models in tandem to detect and track hands. The first model is a palm detector that scans the input image and locates hands using a bounding box. The second is a hand landmark model that generates 2.5D landmarks for the cropped hand bounding box provided by the palm detector^28^. Doing so significantly reduces the need for data augmentation, and the system can focus entirely on producing accurate hand and finger landmarks^24^ (Fig. 4).

To ensure real-time hand tracking, the bounding box from the previous frame is used as input for the current frame, mitigating the necessity to apply the detector across the entirety of each captured frame. In the AR-BBT system, only one hand is needed to move blocks across the box, so only the first will be tracked if multiple hands are detected.

To simulate grabbing a block, the algorithm first estimates the hand's pose and determines whether the thumb and index fingers are closed. This is done by calculating the distance between the fingertips using the Euclidean distance and a preset threshold, which has been adjusted for best results when the hand is approximately 50 cm from the camera. Once the hand's position is known, the system checks whether the participating fingers (i.e., the thumb and index finger) are within the region of a virtual block and whether the fingers are closed (i.e., the distance between the thumb and index finger is less than the preset threshold). If so, the block is considered movable. The block's position is calculated on each frame, and collision checking is performed to prevent the block from passing through the partition divider. Once a block has been moved successfully, the patient earns a point, and the block falls into the respective compartment. Finally, the system includes out-of-bounds checks to keep the blocks within the virtual box's region^25^.

Ensuring precise identification of the patient's hand and fingers during diagnostic procedures is imperative to uphold the reliability and efficacy of the process. While our proposed solution demonstrates encouraging outcomes under optimal operational conditions, our testing has revealed certain constraints embedded in our application, mainly attributed to inherent limitations present on camera hardware, which were nevertheless mitigated as described in the rest of this paragraph, without affecting hand detection or presenting missed successful box moving events. For instance, hand detection can be significantly influenced by several environmental variables encompassing many factors. Firstly, ambient lighting conditions play a pivotal role in the accuracy of hand detection. The intensity, direction, and uniformity of light in the testing environment can profoundly impact the visibility and contrast of the hand against its surroundings. Insufficient or uneven lighting may obscure the hand's features, leading to inaccuracies in detection. When natural lighting is unavailable, employing warm (non-white) illumination lamps positioned away from the camera can still yield satisfactory performance, even in darker environments. Secondly, the complexity of the background within the execution environment presents another challenge. Thus, we avoided intricate or cluttered backgrounds, which could create visual distractions, making it harder for the detection algorithm to distinguish the hand from its surroundings. Additionally, we didn’t use patterns or textures in the background, which may interfere with feature extraction and further complicate the detection process. Finally, the individual characteristics of the user's hands introduce variability that must be accounted for in the detection algorithm. Skin tone, shape, size, and hand orientation can vary significantly among individuals. All these variations present further complexities in hand detection, and this was the main reason we used MediaPipe, which, at least at this time, is the most reliable machine learning-based solution in hand tracking since both models are trained by datasets containing millions of types of hands.

#### An example test run of the implemented system

During the testing, the system displays a note on the top of the bounding frame to indicate which hand it recognized. The hand's identifier remains visible throughout the entire process. Figure 4 demonstrates the system's accurate hand tracking by depicting the process of grasping and moving a virtual block. The AR-BBT performs optimally with cameras that can record at least 60 frames per second and an image quality of at least 720p. Nonetheless, by reducing the resolution to 480p, the system can still function adequately. Changing the target width and height values can be done by modifying the main script file bundled with the application. The only prerequisite for launching the AR-BBT is a functioning Python 3 installation, as the system includes a distinct file containing the necessary libraries and tools. Last, the implementation of the system can be readily expanded to accommodate any additional features that may be needed.

### Subjects

G*Power 3.1.9.7 software for Windows (Heinrich-Heine-Universität Düsseldorf, Düsseldorf, Germany) was employed to conduct an a priori power analysis to determine the appropriate sample size needed to achieve a large effect size with 80% power and an α error probability of 0.05. A nondirectional (two-tailed) analysis was applied. As our approach is innovative and AR-BBT score using machine learning (ML) technology has not been used before for this purpose, in comparison to conventional BBT score, there are no previous studies to determine the actual correlation between these two continuous variables in the population. Pearson’s correlation coefficient (ρ H1) was set at 0.5, indicating a moderate correlation^29^. According to the aforementioned parameters, a total sample size of 29 subjects is needed to achieve the desired statistical power. This study was approved by the Ethical Committee of the University of the Peloponnese (No: 20366/08.09.2022). In compliance with prevailing regulations and the Declaration of Helsinki, all research procedures were rigorously followed. It is also affirmed that informed consent was secured from every participant for study participation, including those presented in Figs. 3 and 4, for publication in an open-access journal.

Our recruitment procedure involved constantly enrolling patients until the necessary sample size was reached. Prior to commencing the trial, we evaluated the research population and assessed the suitability and expenses associated with the recruiting method. The recruiting concentrated on the recommendations of a single physician. We prioritized the cost-effectiveness of the recruiting approach adopted.^30^

Initially, forty-eight subjects were clinically evaluated. The inclusion criteria applied were hemorrhagic stroke pathology, an Ashworth Scale score of 3 or less, and a maximum time since the incident of 6 months. The exclusion criteria were an Ashworth Scale score greater than 3, time since the incident of more than 6 months and the presence of neurological pathologies that affect hand motion (Parkinson’s disease) or musculoskeletal deficits that affect normal finger range of motion (e.g. finger fractures, joint stiffness/pain, arthritis that affect joint motion). Five patients were excluded because of the presence of co-morbidities (Parkinson’s disease), nine because of rheumatoid arthritis at the finger joints and subsequent pain and three because of fingers’ joint stiffness and lack of full range of motion (ROM). Finally, thirty-one patients were included for hand dexterity evaluation with the AR-BBT and regular BBT (twenty males and eleven females). The mean age was 72.22 years (SD = 5.23). The mean time from the diagnosis was 4.49 months (SD = 1.12).

### Clinical examination

All thirty-one subjects included for further evaluation were clinically examined by the same clinician. The Ashworth Scale was evaluated for the flexor muscles of the affected hand (16 right and 15 left hands). The mean score of the Ashworth Scale was 1.96 (SD = 0.89) (Table 1). The passive and active range of finger flexion was normal. The demographic data and the Ashworth scale score are shown in Table 1.

**Table 1:** Demographic data and Ashworth scale scores of the sample.

Item	Stroke patients (mean ± SD)
Total number	31
Gender	
Male	20
Female	11
Age (years)	72.22 ± 5.23
Time from first diagnosis (months)	4.49 ± 1.12
Ashworth Scale score	1.96 ± 0.89

### Patients’ experience questionnaire

Previous studies assessed poststroke patients’ user experience of AR assessment tools^34^. Relative Questionnaire used to assess subjective feelings and experiences of users. In our study we addressed this issue with the following questionnaire (Table 2).

**Table 2:** Relative Questionnaire to assess subjective feelings and experiences of users.

Questions	Answears			
	Strongly disagree	Disagree	Agree	Strongly agree
I was excited				
I learned something new				
I was frustrated				
I was bored				
I felt successful				
I had fun				
I was angry				
I was motivated				
I was challenged by the absence of physical items				
I was fascinated				
I think this tool can support Rehabilitation				

### Clinical procedure

The Augmented Reality BBT laptop is placed on a standard height table (approx. 0.8 m). Each participant is positioned in front of the table on a standard height chair (0.6 m). Blocks are in the compartment of the box adjacent to the assessed hand. The clinician remains next to the subject but outside the camera's field of view to observe the movement of the blocks while the camera only captures the subject's hand. Prior to the clinical procedure, a session specifically focused on familiarizing the patient with the technique was held. The clinician provided a detailed explanation of the application and enabled each patient to undergo five 60-s trials to familiarize themselves with the process. The score of the moved blocks is displayed in the upper-left corner of the screen upon the conclusion of each test. The system prohibits the subject from grasping and working multiple blocks. If the block meets the divider, it vanishes, and the participant must start again with a new block. For the transfer to be considered successful, the subject must separate his/her fingertips and release the block in the designated compartment (Fig. 3).

The clinical procedure lasted 12 months, from the recruitment period until the final evaluation of the thirty-first patient’s assessment with the AR-BBT.

## Results

**Figure 5:**
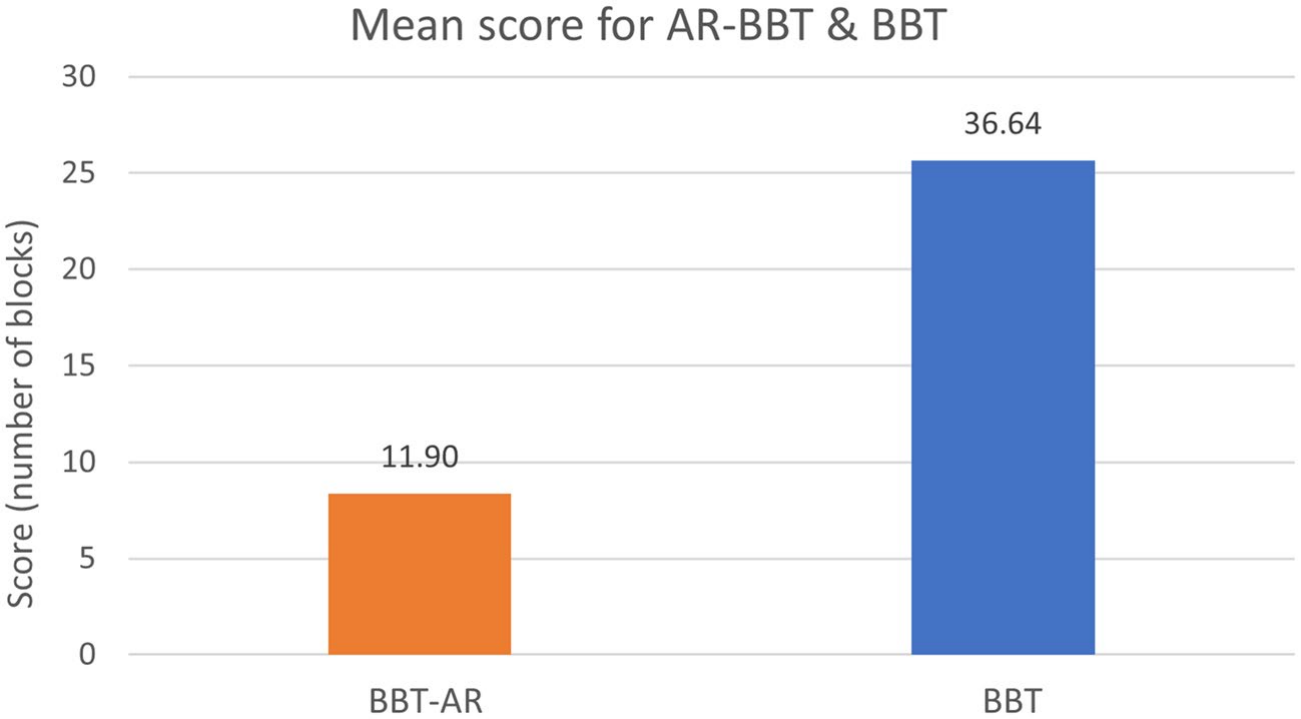
Number of blocks moved successfully (actual score) for the AR-BBT and BBT.

The mean score achieved with the use of the AR-BBT was 11.90 (SD = 2.11), and the BBT mean score was 36.64 (SD = 6.14) (Fig. 5).

**Figure 6:**
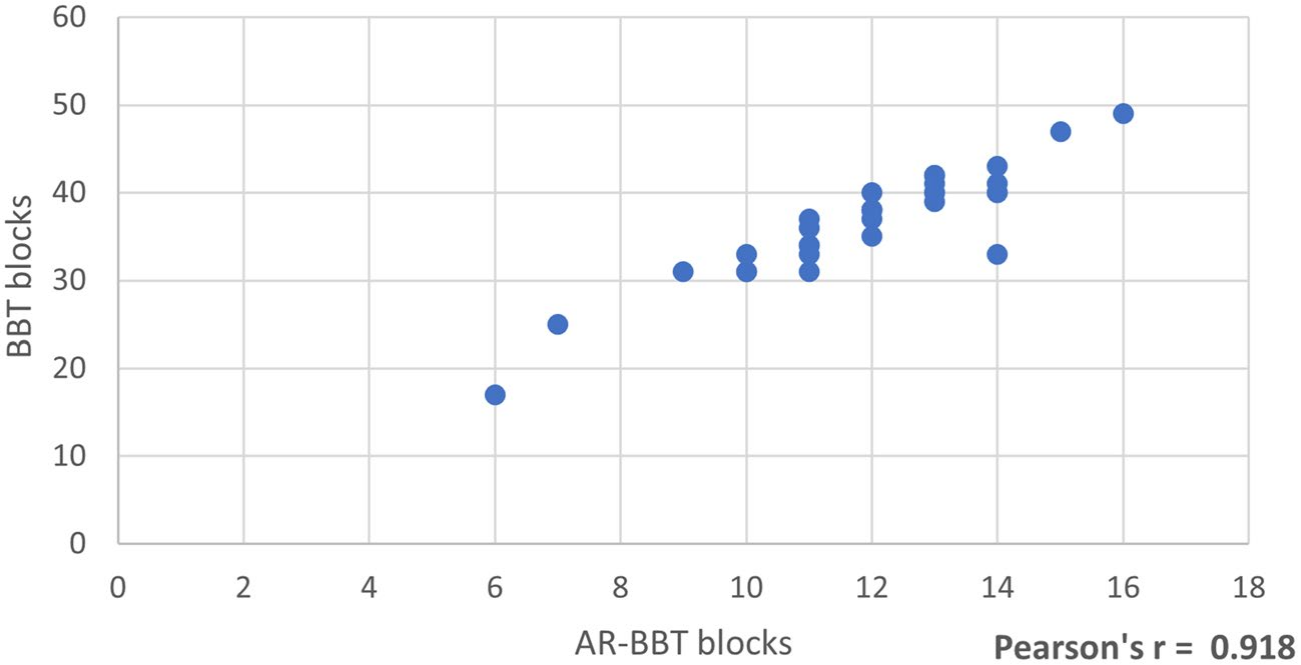
The Pearson Correlation Coefficient (Pearson’s r) was calculated between the scores of the AR-BBT and the traditional BBT. There was a strong and positive correlation (r = 0.918) between the number of blocks moved in the BBT (Y-Axis) and the AR-BBT (X-Axis) on the hemiplegic side.

There was a statistically significant decrease in the number of cubes moved in the AR-BBT compared to the BBT (*p* < 0.001 on the hemiplegic side). The Pearson correlation coefficient (Pearson’s r) was calculated between the scores of the AR-BBT and the traditional BBT. There was a strong and positive correlation (r = 0.918) between the number of blocks moved in the BBT (Y-Axis) and the AR-BBT (X-Axis) on the hemiplegic side (Fig. 6). The Pearson correlation coefficient was classified based on Cohen's criteria (Cohen 1988) as follows: r = 0.10 indicating a minor effect size, r = 0.30 indicating a medium effect size, and r = 0.50 indicating a large effect size.

**Figure 7:**
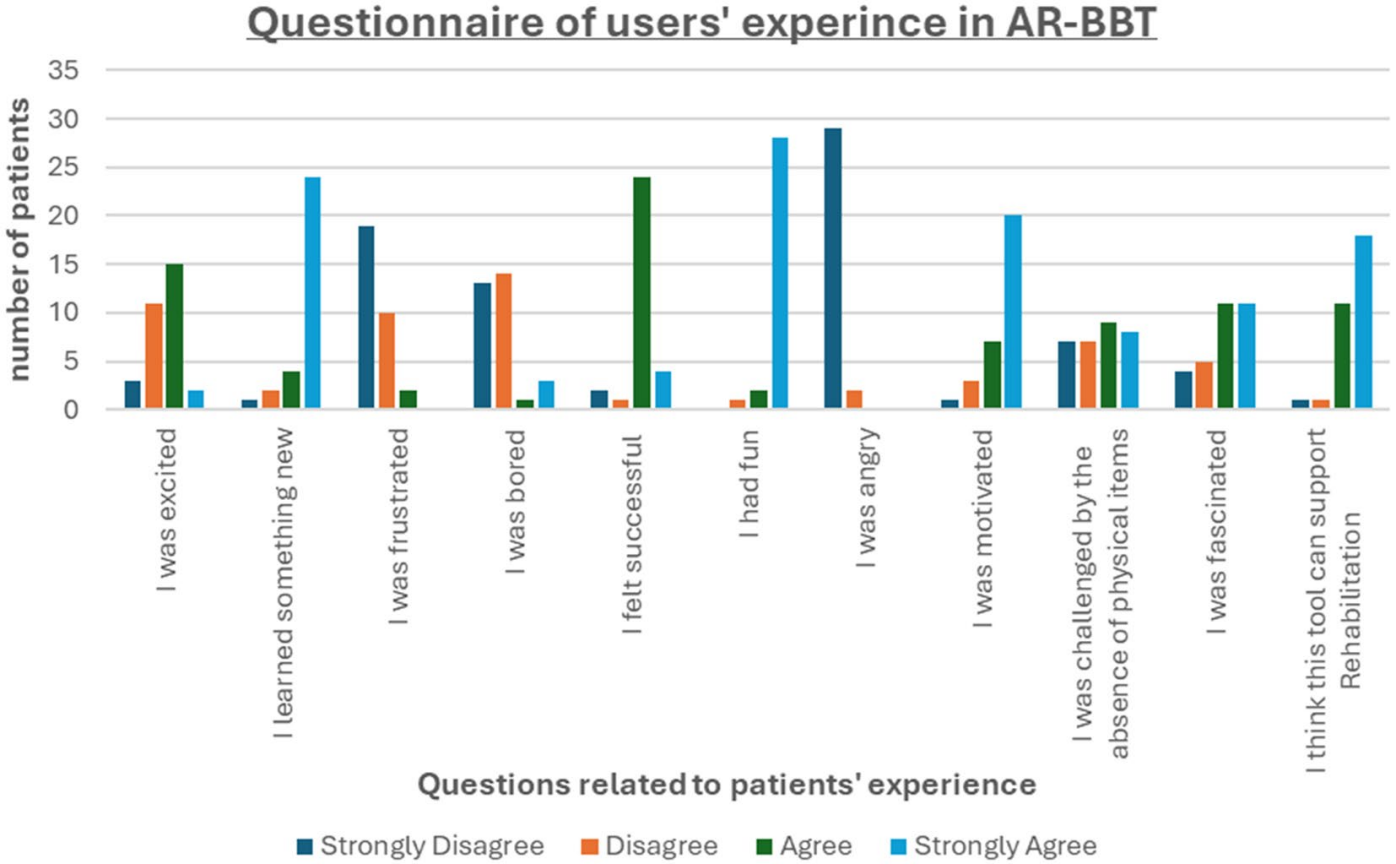
Questionnaire of users' experience in AR-BBT. Patients referred good perception of their experience with augmented reality exercises.

We used the questionnaire shown in Table 2 to assess patients' experience with AR-BBT performance. The results indicated that, overall, the patients had a good perception of their experience with augmented reality exercises (Fig. 7). The 54.8% of respondents agreed or strongly agreed that they were excited about the AR-BBT procedure. 90% agreed or strongly agreed that they learned something new. 93.5% and 87% disagreed or strongly disagreed that they were frustrated or bored, respectively. 90% agreed or strongly agreed that they felt successful, and 96.7% had fun during the procedure. All individuals did not feel angry (100%). Yet 87% agreed or strongly agreed that they felt driven, 70.9% were captivated, and 93.5% believed that AR-BBT may assist rehabilitation processes. 45.2% of respondents disagreed or strongly disagreed that they were challenged by the absence of physical items.

## Discussion

We have replaced the standard BBT with a virtual process, incorporating all the original evaluation instructions into an interactive computer vision experience. We evaluated the system in individuals recovering from stroke to evaluate hand dexterities, and a strong correlation between the traditional BBT and AR-BBT was achieved.

The use of such technologies for Virtual patients' assessment through BBT is a relatively new, but highly intriguing and promising approach in rehabilitation, and only a small number of studies have been published. Their primary goal is to provide an objective assessment of hand dexterity, the automation of the procedure, and the correlation of the virtual setting to the traditional BBT test in Parkinson's disease^22^, spinal cord injury^23^, and post-stroke patients^21,31^. Other studies explored the feasibility of measuring additional clinical data (hand kinematic parameters) via VR-BBT^19^ or investigated the limitations of computer-based BBTs^20,32^.

As stated above, there is currently a limited number of studies that have utilized a virtual version of the box and block test (BBT) for post-stroke hand dexterity assessment, and none of them have done so in an augmented reality environment^33^. To construct and simulate the testing environment, the aforementioned studies require additional equipment that is often costly and reduces the overall portability of the application. Our findings are consistent with VR-BBTs cited in the literature, overcoming the limitations of extra additional and typically costly equipment components while maintaining a strong correlation with conventional, reliable BBTs. Such an AR-BBT could be used as an easy-to-use clinical instrument to assess the hand dexterity of stroke patients.

A number of studies utilized augmented reality (AR) technology to evaluate hand dexterity, but none of them employed the gold standard traditional BBT concept. AR solutions for hand dexterity evaluation show potential for clinical use. Virginia and colleagues conducted a systematic review in 2018 on usability assessments for augmented reality motor rehabilitation solutions. They emphasized the significance of considering users' experiences with such systems in addition to quantitative data from test results to investigate their therapeutic potential fully. Their research confirmed that the most effective approach is utilizing quantitative data and qualitative analytical methods, such as user questionnaires and interviews. Unfortunately, only a limited number of research also explore users' subjective emotions and experiences in AR neurological hand dexterity evaluation examinations. Hossain et al.^34^ evaluated the user experience of augmented reality evaluation tools in poststroke patients. The researchers utilized a Relative Questionnaire to assess participants' subjective sentiments and experiences. Their findings indicated that patients generally viewed their experience in AR tasks positively, with the data from our study agreeing. Similar findings were seen in other studies^35,36^ where individuals initially reluctant about computer games expressed a desire to continue playing after gaining hands-on experience. Cameirao et al.^37^ utilized a self-report 5-point Likert questionnaire to assess patients' experience during the examined task. An average rating of 4.4/5 was reached for overall satisfaction to continue the therapy tasks. In Ganjiwale et al.^38^, participants in the intervention group described the treatment as engaging and entertaining during casual conversations.

Ganjiwale also examined how patient demographics influenced their performance in the trial. A favorable attitude towards utilizing computer systems was crucial in the treatments and helped overcome the age barrier. Children and teenagers are likely to be more receptive to the introduction of computer games due to their inclination to play^39^. The selected studies in their systematic review, however, show that all patients adopted the recommended systems equally, independent of their age group, which contradicts the premise.

Every patient is unique and necessitates tailored treatment that caters to their specific requirements. Most of the virtual solutions found at the literature involve typical movements such as finger flexion/extension, finger abduction/adduction, pinching with object grip and release, and wrist flexion/extension. The physiotherapist's responsibility is to determine the appropriate dosage for each activity.

AR/VR systems can be utilized as supplementary tools to improve dexterity assessment, hand rehabilitation and motor retraining. More specifically, individuals can undergo neuromuscular reeducation using less immersive virtual environments, such as the AR-BBT proposed in our study. Movements acquired in AR/VR settings can typically be applied to real-world tasks and, in certain cases, even extend to untrained tasks. On the other side age and gender do not seem to hinder this process^35^.

A few studies that compared hand rehabilitation in real versus augmented/virtual environments have also indicated AR/VR training advantages. These systems offer interactive task-based activities and visual and auditory feedback on performance, motivating players to enhance rehabilitation intensity. Basic systems like leap motion controller (LMC) and haptic gloves may be included in residential environments. Yet their primary disadvantage compared to our proposed solution is the additional equipment they require.

Despite significant correlations between physical and virtual BBTs in the relative score comparison of affected sides, the number of blocks moved in the BBT by all attendants was significantly higher than the number of blocks moved in the AR-BBT. This is consistent with previous research highlighting the disparity between virtual and real-world assessments. Multiple factors may have contributed to this result. This is initially supported by interactions with virtual reality, where the lack of tactile sensation is a crucial factor influencing the simplicity of interaction and application. Furthermore, depth perception may be difficult with only two-dimensional visual input. To overcome it, Gieser et al.^32^ proposed a virtual BBT comprised of active hand tracking with an immersive VR apparatus, which was administered to healthy individuals to determine whether the subjects' familiarity with VR and computer technologies influenced their performance. Overall, they concluded that it appeared that experience with video games and VR was associated with a superior ability to perform the computer task as the level of video game/VR experience was positively correlated with the total cubes moved in the computer task.

Another explanation for the number of blocks transferred in the AR-BBT being substantially smaller than in the BBT derived from a lack of sensory input. (e.g., mass, collision, and friction). Meli et al. explored the effect of haptic feedback on the performance of the participants in an AR-test inspired by the box and block test^20^. They utilized haptic feedback provided by two different wearable devices, one with 2 degrees of freedom (DoF) and one with 3, to provide sensory inputs to the subjects' fingertips when they touched the block. They identified statistically significant differences in scores—higher scores for both types of haptic feedback compared to no haptic feedback—and attributed the lower scores of VR-BBTs to the absence of sensory inputs.

Frequently, predictive machine-learning systems fail to express a level of confidence in the accuracy of their outputs. Although our grasping detection method detected the grasping motion well using finger joint angles, in some cases the depth camera does not capture it. Therefore, there is limited recognition of the gestures of users compared to a BBT in which various grasping gestures are used. To prevent incorrect predictions from machine-learning models, system users need to be aware of the model's overall precision and the degree of confidence associated with each individual prediction^40^.

In conclusion, the AR-BBT system enables patients to assess their manual dexterity autonomously in a clinical environment or at home without needing the presence of a doctor. It can be installed on any PC or laptop with a web camera without requiring extra equipment or hardware, making it a more cost-effective option than current VR systems or the original BBT. Self-assessment convenience can lead to more frequent evaluations, speeding up the patient's recovery. Furthermore, it enhances remote and long-term monitoring, which is crucial for the treating physician. Augmented reality technology appears to be a valuable tool for evaluating hand dexterity. Every aspect of hand functioning is a complex clinical procedure that relies on the patient's pathology. Further research is needed to create additional hand dexterity testing tools, such as the Sollerman test, utilizing augmented reality (AR) technology. Furthermore, these tests should be studied for their clinical applicability in disorders that impact hand functionality beyond stroke. This might offer significant clinical evaluation tools for both professionals and patients.

## Data Availability

Data underlying the results presented in this paper are not publicly available at this time but may be obtained from the authors upon reasonable request. Please contact Ass. Prof. Georgios Papagiannis (grpapagiannis@yahoo.gr).
